# Resveratrol and Other Natural Oligomeric Stilbenoid Compounds and Their Therapeutic Applications

**DOI:** 10.3390/plants12162935

**Published:** 2023-08-14

**Authors:** Cosmina-Gabriela Duta-Bratu, George Mihai Nitulescu, Dragos Paul Mihai, Octavian Tudorel Olaru

**Affiliations:** Faculty of Pharmacy, “Carol Davila” University of Medicine and Pharmacy, Traian Vuia 6, 020956 Bucharest, Romaniaoctavian.olaru@umfcd.ro (O.T.O.)

**Keywords:** polyphenolic compounds, anticancer activity, cardiovascular health, resveratrol, piceatannol, pterostilbene

## Abstract

The use of natural compounds as an alternative to synthetic molecules has become a significant subject of interest in recent decades. Stilbenoids are a group of phenolic compounds found in many plant species and they have recently gained the focus of a multitude of studies in medicine and chemistry, resveratrol being the most representative molecule. In this review, we focused on the research that illustrates the therapeutic potential of this class of natural molecules considering various diseases with higher incidence rates. PubChem database was searched for bioactivities of natural stilbenoids, while several keywords (i.e., “stilbenoids”, “stilbenoid anticancer”) were used to query PubMed database for relevant studies. The diversity and the simplicity of stilbenes’ chemical structures together with the numerous biological sources are key elements that can simplify both the isolation of these compounds and the drug design of novel bioactive molecules. Resveratrol and other related compounds are heterogeneously distributed in plants and are mainly found in grapes and wine. Natural stilbenes were shown to possess a wide range of biological activities, such as antioxidant, anti-inflammatory, antihyperglycemic, cardioprotective, neuroprotective, and antineoplastic properties. While resveratrol is widely investigated for its benefits in various disorders, further studies are warranted to properly harness the therapeutic potential of less popular stilbenoid compounds.

## 1. Introduction

The secondary metabolites, like alkaloids, terpenoids, or phenolics, are organic compounds that are not directly involved in the growth, development, or reproduction of an organism, but they play important roles in various interactions in response to environmental stimuli, such as stress, competition, or signaling [1,2]. Stilbenes are plants’ secondary metabolites that play important roles in plants, particularly in their defense mechanisms against pathogens such as fungi, bacteria, or viruses and help plants combat oxidative stress caused by UV radiation, pollutants, and reactive oxygen species or abiotic stress, such as drought, temperature extremes, and mechanical damage [3,4]. Some stilbenes are involved in allelopathy, inhibiting the growth and development of nearby plants in their competition for resources and survival [5].

A wide variety of natural stilbene derivatives have been identified and their structure ranges from monomers to octamers possessing different substituents at various positions, like glycosyl, hydroxyl, methyl, or isopropyl groups [6]. Some notable examples of stilbenes include resveratrol, pterostilbene, piceatannol, and viniferins [5]. Among the stilbene compounds, resveratrol is one of the most well-known and widely studied due to its wide range of biological activities, such as antioxidant, anti-inflammatory, and stimulant of adipocytes lipolysis [7].

The distribution of natural stilbenes in the plant kingdom is limited. However, they can be found in taxonomically distant species within the Embryophyta phylum (land plants), from less complex species to the most advanced angiosperms. Stilbenes have been isolated from Bryophytes (Marchantiales order), Monilophytes (Ophioglossales and Polypodiales orders), gymnosperms (Gnetales and Pinales orders) and angiosperms (Magnoliophyta division, in both Monocots and Eudicots). The 2-arylbenzofurans are found in only seven known families: Corsiniaceae, Gnetaceae, Melanthiaceae, Stemonaceae, Moraceae, Fabaceae, and Vitaceae [8].

Many studies about the therapeutical effects stilbenes have been published to date, which can lead to the evidence of the potential these molecules might have and to the interest scientists have given to them. The interest and research focus on stilbenes stem from their natural occurrence, diverse pharmacological activities, and potential applications in promoting human health and well-being.

This review aims to provide a comprehensive overview of the natural oligomeric *trans*-stilbenoid compounds and their therapeutic applications. It illustrates the natural sources’ diversity and structural characteristics of these compounds, highlighting their bioavailability and mechanisms of action. Furthermore, it explores the emerging evidence for their therapeutic potential in various disease conditions, discussing preclinical and clinical studies that support their efficacy and safety profiles. The PubChem database was queried using structures of stilbenoid compounds to search for relevant bioactivity data. PubMed was searched for relevant studies involving the therapeutic potential of natural stilbenes using several keywords, such as “natural stilbenoids”, “oligomeric stilbenes”, “bioactive stilbenes”.

## 2. Chemistry of Stilbenoids

The stilbenoids are a group of natural phenolic compounds sharing a stilbene backbone structure and different substituents on the rings [9]. The stilbene scaffold consists of two benzene rings joined by an ethylene segment [10]. There are two isomeric forms of 1,2-diphenylethylene: (*E*)-stilbene (*trans*-stilbene), which is not sterically hindered, and (*Z*)-stilbene (*cis*-stilbene), which is less stable because of the steric interactions between the aromatic rings [11]. Dihydrostilbenoids are related compounds known also as bibenzyls, where the double bond is replaced by a simple one [12].

Based on their chemical structure, the stilbenoids can be classified into five major groups: simple stilbenes, prenylated and geranylated stilbenes, 2-phenyl-benzofuran derivatives, carbon substituted stilbenes that do not belong to the prenylated and geranylated stilbenes group, and various other structures [13]. Stilbenoids exist as monomers or oligomers. They may also be found free phenolic derivatives (aglycone) or conjugated as glucosides [9]. 

**Figure 1:**
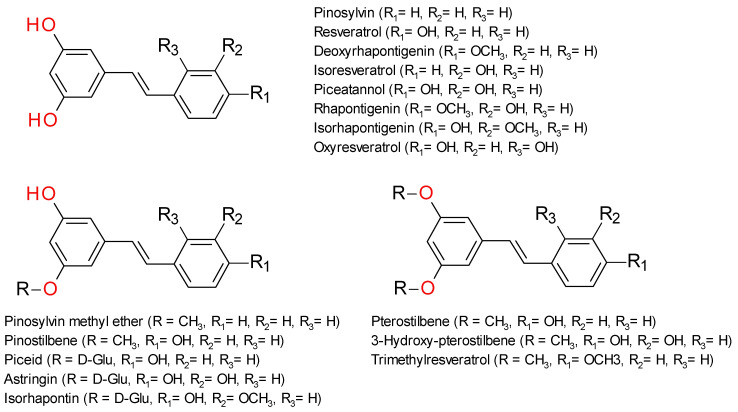
Structures of representative stilbenoids compounds chemically classified as simple stilbenes.

The first class, frequently referred as simple stilbenes, shares the 1,2-diphenylethylene scaffold without any additional carbon–carbon bonds. The benzene rings are substituted in various positions with hydroxyl, methoxy, or glycosyl groups [14]. These derivatives exhibit various substitution patterns on the aromatic rings that can greatly influence the chemical properties and biological activities. The majority of the stilbenes in this group are 3,5-dihydroxy substituted, while the second aromatic ring may have hydroxyl or methoxy groups. Figure 1 presents the structures of the main stilbene derivatives based on the substitution of the 3,5-dihydroxy groups.

The second group is represented by the prenylated and geranylated stilbenes, derivatives with additional carbon–carbon bonds into the 1,2-diphenylethylene scaffold. Prenyl and geranyl groups are derived from isoprenoid precursors and consist of five and ten carbon atoms, respectively. These groups are often added to the stilbene core through enzymatic reactions in plants, fungi, and some microorganisms [15]. The addition of one or several moieties can occur at various positions on the phenyl rings leading to compounds with unique chemical properties. There are possible subsequent modifications of the prenyl group, such as cyclization and hydroxylation [16].

Chiricanines A–E are five prenylated stilbenes found in *Lonchocarpus chiricanus* (Leguminosae) together with longistylines C and D, compounds that are also found in the root and the bark of *Lonchocarpus violaceus*. The structures of these stilbenes contain on or two prenyls fragments [17,18]. In chiricanines B and D, the prenyl cyclizes to form a dimethylchromene ring, whereas the prenyl of chiricanines E forms a hydroxyl-substituted dihydrobenzofuran ring with the double bond in *cis* configuration [19].

Mappain is cytotoxic stilbene from *Macaranga mappa*, a derivative of piceatannol that is substituted with a prenyl and a geranyl group. It is considered the biogenetic precursor of vedelianin and a homologue of schweinfurthin C, a di-geranyl stilbene from *Macaranga schweinfurthii* [20,21]. Schweinfurthins A, B, and D are structurally similar hexahydroxanthene stilbenes isolated from *Macaranga schweinfurthii* [22].

**Figure 2:**
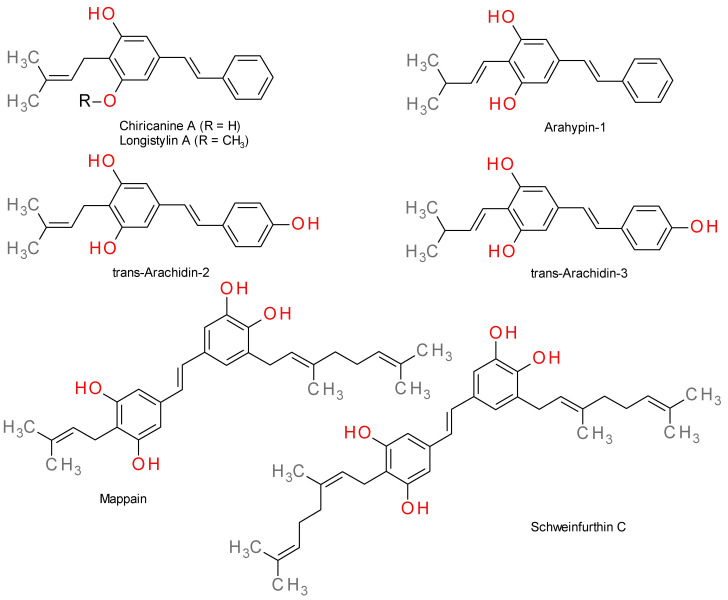
Structures of prenylated and geranylated stilbenes.

Some examples of prenylated stilbenes like chiricanine A, arahypin-1, *trans*-arachidin-2, *trans*-arachidin-3, and longistylin A [13,23] are presented in Figure 2 together with representative geranylated stilbenes.

## 3. Vegetal Sources

Stilbenes and stilbenoids are biosynthesized through the phenylpropanoid pathway [24]. The first step in the biosynthesis of the stilbene backbone involves, in a single reaction, the combination of three malonyl-CoA and one CoA-ester of a cinnamic acid derivative. The resulted polyketide is then used in the production of flavonoids or stilbenoids, taking into consideration the enzyme involved: chalcone synthase or stilbene synthase, respectively [25,26].

The stilbene synthase is not expressed in all plant species, so, the distribution of natural stilbenes in the plant kingdom is limited. However, they can be found in taxonomically distant species within the Embryophyta phylum (land plants), from less complex species to the most advanced angiosperms (Table S1). Stilbenes have been isolated from Bryophytes (Marchantiales order), Monilophytes (Ophioglossales and Polypodiales orders), gymnosperms (Gnetales and Pinales orders), and angiosperms (Magnoliophyta division, in both Monocots and Eudicots). The 2-arylbenzofurans are found in only seven known families: Corsiniaceae, Gnetaceae, Melanthiaceae, Stemonaceae, Moraceae, Fabaceae, and Vitaceae [27].

The main natural source of stilbenoids is represented by *Vitis vinifera* L. species, which belongs to the plant family Vitaceae. Over 60 stilbenoids can be found in this species as monomers, such as *trans*-resveratrol or piceatannol, and as oligomers, which are usually in their *trans* configuration € [8].

There are 16 genera within Vitaceae and only eight of them are known to have stilbenoids identified as chemical constituents: *Vitis*, *Ampelopsis*, *Cayratia*, *Cissus*, *Cyphostemma*, *Rhoicissus*, *Muscadinia*, *Parthenocissus*. The most economically important remains *Vitis vinifera*, but Americans use the varieties *V. berlandieri*, *V. riparia*, and *V. rupestris* for growing wine and the grapes industry as well. In the United States, the *V. labrusca* cv. Concord is particularly important, serving as the main grape used in juice, Passover wine, and the peanut butter and jelly sandwich. The Asian species, *V. amurensis*, *V. coignetiae*, and *V. thunbergii*, are known to have been historically used as medicinal plants for a range of diseases, such as inflammation and hepatoprotection [8].

Stilbenes can be found in *Vitis vinifera* varieties as constitutive compounds of the lignified organs (roots, seeds, stems, canes, ripe cluster stems) and as induced substances (in leaves and berries). They mainly act as phytoalexins in the mechanisms of grape resistance against pathogens. Resveratrol and piceid can be found in grape products and the greatest concentration of resveratrol found in the skins is in glycosidic forms. On the other side, pterostilbene is detected in low levels in healthy and immature grape berries [28].

In seeds, only *trans*- and *cis*-resveratrol was detected, whereas resveratrol, piceid, resveratroloside, and astringin, both in *cis*- and *trans*- isomeric forms, were found in the grape cell suspension cultures. Moreover, three resveratrol diglucosides, *cis*- and *trans*-resveratrol 3,5-*O*-β-diglucoside and *trans*-resveratrol 3,4′-*O*-β-diglucoside, were isolated together with a resveratrol triglucoside, *trans*-resveratrol 3,5,4′-*O*-β-triglucoside [29,30].

A study performed on a German commercial white wine (Riesling) showed the existence of another nine stilbenes, besides resveratrol and piceid. These compounds included the monostilbene 2,4,6-trihydroxyphenanthrene-2-*O*-glucoside, two isomeric resveratrol-2-*C*-glucosides and also *cis*- and *trans*-epsilon-viniferin diglucoside, pallidol glucoside, and pallidol diglucoside, which were found at very low levels (<0.05 mg/L) [31].

From French commercial red wines, *trans*-α-viniferin, parthenocissin A, and pallidol have been isolated [32] and the Brazilian red wines contain *trans*-astringin, *trans*-piceid, *trans*-resveratrol, *cis*-resveratrol (5 times more than the trans- form), epsilon-viniferin, and *trans*-delta-viniferin [33].

Stilbenes can be found in minor contents in *Vaccinium* berries such as *V. myrtillus* (bilberries: 0.02–0.77 µg/g dry weight), *V. elliotti* (blueberries: 0.45 µg/g dry weight) *V. macrocarpon* (cranberries), *V. vitis-idaea* (lingonberries), *Morus* (mulberries), *Fragaria x ananassa* (strawberries) [34].

Although not at high level, studies have shown that piceid is in a higher concentration than resveratrol in some vegetable food: rhubarb, banana, guava, leech, pineapple, apple, peach, passion fruit, pears [35,36,37,38].

Reasonable concentrations of piceid were found in almonds, the highest ones being in the blanch water (6.33–8.43 µg/100 g), which also contains piceatannol and oxyresveratrol (0.91–2.55 µg/100 g) and in the skin (0.15–0.22 µg/100 g) [39]; moreover, gnetol and resveratrol dimers, such as gnetin C and its glucosides, gnemonosides A, C, and D [40].

However, wine remains the most important source of stilbenes and it represents 98.4% of the intake, followed by grape berries and grape juice (1.6%), while peanuts and other berries would contribute less than 0.01% [41].

Phenolic compounds in red wine have different sources, the largest one being found in black grapes. The main factor that influences the concentration of polyphenols in red wine is the vinification technique (the maceration–fermentation stage) [42]. During the fermentation process, the wine is enriched in polyphenols, depending on the tyrosol that is released in wine. Tyrosol is a compound produced by tyrosine (by the action of tyrosine decarboxylase) or a *para*-coumaric acid precursor [43].

## 4. Bioactive Stilbenes

Since the studies performed on the French Paradox, stilbenoid compounds, in particular *trans*-resveratrol and its glucoside, have received increasing scientific attention. Resveratrol possesses a wide range of biological properties, among them antioxidant, cardioprotective, neuroprotective, anti-inflammatory, and anticancer activities [44,45]. Resveratrol exhibits unfavorable pharmacokinetic characteristics, such as short half-life, rapid clearance, and low bioavailability. The low bioavailability is attributed to its extensive metabolism by phase II enzymes [46]. The co-administration of resveratrol with piperine, an established glucuronidation inhibitor, significantly increased the in vivo bioavailability of resveratrol [47].

The relationship between chemical structure and biological activity has been widely studied and it is still under debate. The stilbene scaffold was subject to many synthetic molecules due to its demonstrated properties: anti-cancer [46], anti-inflammatory [48], antimicrobial [49], antifungal [50], neuroprotective [51]. This is how some molecules that nowadays are in use were designed: toremifene, raloxifene, or tamoxifene [52].

Resveratrol is the most acclaimed stilbene demonstrated to have many beneficial properties [53]. Due to its low quantities in natural resources and low bioavailability, researchers have focused on the key elements of the resveratrol’s structure to design synthetic molecules [54]. Evaluation of pro-oxidant action of different stilbenes in the presence of copper revealed the importance of the hydroxyl group at the 4′ position [55].

Currently, more than 162 clinical studies have been published on the online PubMed database (http://www.ncbi.nlm.nih.gov/pubmed (accessed on 12 June 2023), of which 95% appeared in the last decade. Interestingly, some of the resveratrol clinical trials registered at www.clinicaltrials.gov (accessed on 12 June 2023) are unpublished although most studies are completed [56]. A list of completed clinical trials performed on stilbene derivatives is presented in Table 1.

### 4.1. Positive Effects on the Cardiovascular System

Clinical trials have looked into the protective and curative effects of resveratrol against a number of diseases and disorders, and both clinical and preclinical data have shown that this molecule has numerous targets and that it can modulate many signaling molecules including: Wnt, nuclear factor –κB, cytokines, caspases, Notch, matrix metalloproteinases (MMPs), 5′-AMP-activated protein kinase (AMPK), intercellular adhesion molecule (ICAM), vascular cell adhesion molecule (VCAM), sirtuin type 1 (SIRT1), tumor necrosis factor α (TNF-α), peroxisome proliferator-activated receptor-γ coactivator 1α (PGC-1α), insulin-like growth factor 1 (IGF-1), insulin-like growth factor-binding protein (IGFBP-3), RAS association domain family 1 isoform A (RASSF-1α), pAkt, vascular endothelial growth factor (VEGF), cyclooxygenase 2 (COX-2), nuclear factor erythroid 2-like 2 (Nrf 2), and Kelch-like ECH-associated protein 1 [57,58]. Resveratrol has the potency of reducing brain natriuretic peptide (BNP) and improving ventricular function, being administered in a stable case of angina pectoris [59].

Isorhapontigenin is a methoxylated analogue of resveratrol, which is well known also for its anti-platelet activity. Isorhapontigenin possesses greater oral bioavailability than resveratrol. Ravishankar et al. found that isorhapontigenin selectively inhibits ADP-induced platelet aggregation, which is predominantly mediated via the P2Y_12_ receptor, with an IC_50_ of 1.85 µM, although it displayed marginal inhibition on platelet aggregation induced by other platelet agonists at 100 µM. This compound also inhibited integrin αIIbβ3, but no effect was observed on α-granule secretion [60]. Rhapontigenin and desoxyrhapontigenin, two related stilbenes isolated from the rhizomes of *Rheum undulatum*, inhibited the platelet aggregation induced by arachidonic acid.

Panxia Wang et al. demonstrated that isorhapontigenin also contributes to the reduction of the toxicity of doxorubicin. In this study, it was shown that the administration of isorhapontigenin 30 mg/kg/day, intraperitoneally, 3 weeks, significantly protected against doxorubicin-induced cardiotoxicity in mice. In addition, this molecule increased doxorubicin-caused repression in yes-associated protein 1 and the expression of its target genes in vivo and in vitro. Moreover, the inhibition of yes-associated protein 1 blocked the protective effects of isorhapontigenin on doxorubicin-induced cardiotoxicity [61].

The utility of resveratrol is also to lower the toxicity of chemotherapeutic drugs, such as the cardiotoxicity of doxorubicin, by decreasing apoptosis and increasing autophagy in cardiomyocytes. These effects were accompanied by an inhibitory effect on the E2F1/mTORC1 and E2F1/AMPKα2 pathways [62].

At this moment, a study about the use of a combination therapy with nicotinamide riboside and pterostilbene in atherosclerosis is ongoing. Its main purpose is to investigate if this combination can inhibit neurodegeneration and thereby, delay disease development, increase survival, and improve quality of life in atherosclerosis [63].

### 4.2. Anti-Inflammatory Activity

Grape extract is a major source of resveratrol and has been used alone and in combination with pure resveratrol to reduce inflammatory processes. In a placebo-controlled trial, both grape extract and grape extract enriched with resveratrol were administered and the inflammatory cytokines CCL3, IL-1β, and TNF-α were found to be significantly reduced in the peripheral blood mononuclear cells (PBMCs) [64].

Resveratrol inhibits COX-2 and aromatase expression in the eutopic endometrium and so it can be used in dysmenorrhea and pain [65]. Several hydroxylated resveratrol analogues, like piceatannol, have shown selectivity as COX-2 inhibitors, displaying potency comparable to that of the clinically approved celecoxib, but the methoxylated analogues demonstrate limited inhibition of COX-2 activity and lack specificity towards COX-2 [66].

The anti-inflammatory effect of pterostilbene has been studied both in vitro and in vivo. In vitro studies showed that pterostilbene acts through different mechanisms, including the downregulation of cyclooxygenase-2 (COX-2) and inductible nitric oxide synthase (iNOS) levels and also the blockage of the NF-κB signaling, thus suppressing the pro-inflammatory cytokines expression and inhibiting NO production [48,67,68,69]. In vivo, pterostilbene suppressed the NF-κB and AP-1 activity, resulting in inhibiting the activity of COX-2 and iNOS [70].

Pawhuskins, a group of prenylated stilbenes, were extracted from the purple prairie clover (*Dalea purpurea*) and subjected to testing against opioid receptors. Among them, Pawhuskin A demonstrated the highest potency and exhibited competitive antagonism, specifically targeting the KOP receptor [71].

### 4.3. Effects on Diabetes

A study performed in 2013 came with new information and new evidence about resveratrol’s antihyperglycemic effect [72]. Until 2013, only two human studies had been completed to investigate the potential of resveratrol in type 2 diabetes patients; both of them reported a modest reduction in blood glucose levels, while one showed no effect on insulin level [73,74]. The results of this study showed that resveratrol supplementation at a dose of 1 g/day had a remarkable effect in lowering glucose levels and improved other metabolic parameters in humans with type 2 diabetes [72].

According to the results of this study, it may be suggested that short term supplementation with a moderate to high dose of resveratrol (1 g/day) may help to markedly lower blood glucose levels. Furthermore, a lower dose of resveratrol may be administered once blood glucose levels are normalized. A lower dose after 45-day treatment with 1 g resveratrol may eliminate or minimize, if any, resveratrol-related toxicity in the long term. Notable was the fact that the control group which was on standard diabetic treatment alone had an increase in alkaline phosphatase (ALP) levels when compared to the baseline values [72].

Piceatannol is a known AMPK activator and has shown its effect on suppressing the rises in blood glucose levels at early stages and improving the impaired glucose tolerance at late stages in mice. This makes the compound able to prevent and cure type 2 diabetes and become an antidiabetic phytochemical [75].

A study performed in 2013 focused on the inhibitory potential effect of resveratrol and ε-viniferin on D-glucose uptake into porcine jejunal and ileal enterocytes, demonstrating that both polyphenols have the ability to have positive effects, but ε-viniferin exhibited the strongest impact [76].

ε-viniferin has proven positive effects concerning insulin resistance in obesity. This stilbene could inhibit in vitro the activity of α-amilase (IC_50_ = 793.64 ± 0.18 µM) and α-glucosidase (IC_50_ = 23.98 ± 1.00 µM), both enzymes being part of the carbohydrates catabolism [77].

Liu et al. induced type II diabetes on rats and administered ε-viniferin (30 or 60 mg/kg bw) for 8 weeks. Thus, a decrease was observed in fasting blood glucose, triglycerides, total cholesterol, and LDL-cholesterol levels [78].

### 4.4. Neurodegenerative Diseases

Attention was paid on the effects stilbenes may have concerning Alzheimer’s disease. A study conducted by Freyssin et al. was based on the beneficial effects *trans*-resveratrol, *trans-ε*-viniferin, gnetin C, miyabenol C, *trans*-piceid, pterostilbene, piceatannol, and astringin may have in this condition. As a result, some of these stilbenes (gnetin C, *trans*-piceid, piceatannol) had been described only for their in vitro effects up to this study; some others (*trans-ε*-viniferin, pterostilbene) had been studied mostly in in vitro experiments, while in vivo studies remain rare and, still, *trans*-resveratrol remains the mostly discussed stilbene of all of them [79].

Resveratrol has been shown to improve mitochondrial efficiency and muscle activity by activating AMPK, while the levels of SIRT1 and PGC-1α are increased, together with the citrate synthase activity [80].

The sirtuins are a family of proteins categorized as class III histone deacetylases [81]. SIRT1 is postulated to be one of the crucial targets modulated by resveratrol. Preclinical studies suggest that the activation of SIRT1 may contribute to its beneficial effects [82]. For instance, resveratrol has beneficial effects in Parkinson’s disease and Huntington’s disease by inducing SIRT1 activation [83]. Resveratrol also produces many pharmacological effects by activating SIRT1 in both obese and healthy individuals [80,84,85].

Pterostilbene is another compound of this family that exerts its effects through a variety of mechanisms. Pterostilbene shows an anti-tumor effect by regulating a variety of signal pathways and it plays a neuroprotective role by improving and reducing the volume of cerebral infarction, inhibiting apoptosis, and protecting the integrity of the blood–brain barrier. In addition, this molecule shows antioxidant, anti-inflammatory, hypoglycemic, lipid-lowering, antifungal, antiviral, and antipsychotic activities [86].

ε-viniferin possesses positive effects on ROS generation and oxidative stress, more particularly on SIRT3. It was shown that ε-viniferin exhibits consistent cytoprotection in different cell models of Huntington disease and also prevents mitochondrial dysfunction and promotes mitochondrial biogenesis. Furthermore, ε-viniferin has an impact on Alzheimer’s disease by different mechanisms, including the induction of disaggregation of aggregated full-length Aβ42 peptide and the reduction of ROS generation [87].

### 4.5. Malignancies

A growing number of studies reported the antitumor properties of stilbenoid compounds. For instance, Yisi Luo et al. studied the effect of isorhapontigenin on cancer stem cells. This stilbene could inhibit stem cell-like phenotypes and invasiveness of human bladder cancer by attenuation of expression of CD44 but not SOX-2, at both the protein transcription and degradation levels. In addition, further studies showed that isorhapontigenin induced miR-4295, which specifically bound to 3′-UTR activity of *usp28* mRNA and inhibited its translation and expression, while miR-4295 induction was mediated by increased Dicer protein to enhance miR-4295 maturation [88]. Isorhapontigenin has also been reported to have other significant pharmacological effects, such as antioxidant [89] and anti-inflammatory properties [90].

Rhapontigenin was shown by Kim et al. to exhibit antitumor activities in a breast cancer model by suppressing cell migration and invasion through PI3K-dependent Rac1 signaling inhibition [91]. A more recent study highlighted that the stilbenoid glucoside rhaponticin inhibited benzo[a]pyrene-induced lung carcinogenesis in mice at 50 mg/kg bw, improving histopathological alterations and specific biomarkers levels [92]. Moreover, rhaponticin suppressed an osteosarcoma cell-based model by inhibiting the PI3K-Akt-mTOR pathway [93] and tongue squamous cell cancer aggressivity through inhibition of HIF-1α post-transcriptional activity [94].

Pterostilbene was shown to inhibit in vitro the proliferation of a variety of tumor cells, including stomach, lung, liver, oral cavity, pancreas, lymph, colon, prostate, breast, melanoma, leukemia, and myeloma cells. A recent study performed by Shin et al. highlighted that pterostilbene has superior efficacy and bioavailability compared to resveratrol in models of cervical cancer. Pterostilbene inhibited at 10 and 20 µM concentrations the growth and migration of cervical cancer cells, such as HeLa., by inducing cell cycle arrest (through downregulating cyclins E1 and B1 expression) and apoptosis (through activation of caspase-3 and caspase-9, downregulation of Bcl-2 and Bcl-XL, and inhibition of MMP-2 and MMP-9 expression). In vivo, pterostilbene inhibits tumor occurrence and metastasis and showed almost no toxicity. Pterostilbene inhibited tumor growth and tumorigenesis in animal models of colorectal, liver, skin, and brain cancer [95,96,97,98].

Gnetin C is a resveratrol dimer found in grapes and the melinjo plant and has been reported to possess many biological properties, including anti-inflammatory and anticancer properties [99]. In addition, this molecule has shown no toxicity in humans [100]. Gnetin C acts through MTA1/ETS2-mediated mechanisms in prostate cancer and shows significant MTA1-mediated inhibitory effects on cell viability, colony formation and migration, and induces cell cycle arrest and cell death at 25 and 50 µM concentrations. Interestingly, inhibition of MTA1 by 25 µM gnetin C was comparable with the effects of 50 µM pterostilbene and resveratrol [101].

In a pre-clinical study, comparative evaluation of the in vivo efficacy of gnetin C, resveratrol, and pterostilbene in the treatment of prostate cancer led to the conclusion that gnetin C is more potent than resveratrol and pterostilbene, gnetin C having the same antitumor effects as the other two stilbenes in a two-fold lower dose. Moreover, gnetin C was more efficient compared to the other stilbenes at the same dose (50 mg/kg bw). The same study showed that gnetin C exerts its antitumorigenic effects by inhibiting proliferation, angiogenesis, and promoting apoptosis by downregulating MTA1 [102].

Piceatannol was also shown to exhibit significant potential as an anticancer agent, attributed to its notable pro-apoptotic properties and ability to inhibit the proliferation and metastasis of various cancer cell types. This includes lymphoma, leukemia, breast cancer, prostate cancer, and colon cancer, among others [103].

A study published by Yu et al. in 2013 revealed that resveratrol dose-dependently inhibits TRPA1 (Transient Receptor Potential Ankyrin 1) activation by allyl isothiocyanate (AITC), a well-established TRPA1 agonist (pungent compound found in wasabi, horseradish and mustard oil) [104]. TRPA1 is a non-selective calcium channel, member of the TRP superfamily which serves as a sensor for oxidative stress and noxious stimuli [105]. In 2015, Nalli et al. assessed the modulatory activity of pterostilbene, pinosylvin methyl ether, and synthetic stilbenes on TRPA1, in comparison with resveratrol. Unlike resveratrol, pterostilbene and pinosylvin methyl ether show both TRPA1 agonistic (EC_50_ 3.6 and 3.5 µM) and inhibitory (IC_50_ 7.4 and 6.9 µM) activities, while resveratrol only blocked the channel activation, with a 19.9 µM potency. Moreover, several newly synthesized stilbenoids were more potent than the natural derivatives [106]. Previous studies showed that TRPA1-dependent intracellular calcium influx enhances lung and breast cancer cell lines resistance to chemotherapy by up-regulating antioxidant defense and pro-survival mechanisms [107]. On the other hand, activation of TRPA1 mediated by reactive oxygen species in glioblastoma and neuroblastoma cell lines leads to a persistent increase in intracellular calcium concentrations which promotes mitochondrial dysfunction and caspase-3-dependent apoptosis [108]. Therefore, further studies are warranted to explore the potential implications of TRPA1 modulation by natural stilbenoids in their antitumor activities in breast, lung, and brain cancers.

### 4.6. Other Implications

Formulations of resveratrol have also been used for many other diseases. For instance, in healthy smokers, after the resveratrol was administered, there was noticed a reduction of the levels of CRP and triglycerides, as well as the increase of the total antioxidant level [109]. Due to its beneficial effects, a resveratrol containing gel formulation has proven to be useful in acne vulgaris. For this dermatological effect, a clinical trial comprising 20 subjects, a 60-day treatment with resveratrol was performed and it resulted in a 54% mean reduction in the global acne grading system score as compared with 6% on the vehicle treated side of the face [110].

Stilbenes represent a subject of interest also for their potential effects on COVID-19. Resveratrol, pterostilbene, pinosylvin, and piceatannol were studied using computational tools for their potential to act as an inhibitors of the complex formed between the spike (S1) protein from the novel coronavirus and human angiotensin-converting enzyme 2 (ACE2). The results showed that the level of selectivity of resveratrol was remarkably higher compared to other stilbenoids and this potentially strong interaction should be further studied using in vivo and in vitro approaches [111].

## 5. Conclusions

The stilbenoid compounds are a large group of molecules that are alike and different at the same time from their chemical structures to their natural sources and therapeutical activities. Stilbenoids are spread throughout the plant kingdom and their distribution is rather heterogeneous. The main source of stilbenes is represented by grapes and wine, especially red wine. At a lower level, these molecules can also be found in peanuts, blueberries, cranberries, strawberries, and other plants.

Most studies published to date are based on the therapeutical effects of resveratrol due to its beneficial health properties, such as anti-inflammatory, cardioprotective, antioxidant, neuroprotective, and anticancer. This molecule represents a real interest in the pharmaceutical and medical domains and because of its demonstrated effects, many other stilbenes have been investigated, in comparison with resveratrol and not only.

The stilbene family has been lately investigated more for the compounds’ potency of alleviating the symptoms and the causes of different types of cancer and many studies are still ongoing. Some of the molecules that were investigated for their antineoplastic potential are isorhapontigenin, rhapontigenin, rhaponticin, pterostilbene, gnetin C, and piceatannol.

The results of the investigation of stilbenes during the years made the scientists take these compounds into consideration for their possibility of lowering the toxicity of some drugs, resveratrol and isorhapontigenin being able to reduce the toxic effects of doxorubicin.

The importance of stilbenes as bioactive molecules is also highlighted through the studies of the effects these molecules may have against the new pandemic virus, COVID-19, and as a result, between resveratrol, pinosylvin, and piceatannol, resveratrol has been shown to be the most selective and effective stilbene.

To this day, resveratrol remains the most widely studied stilbenoid compound. However, a growing body of emerging evidence supports the beneficial biological activities of the less common stilbenoids. Therefore, further research is warranted to evaluate the beneficial health effects of both resveratrol and less known stilbenoid compounds. As a future perspective, the interaction between natural and synthetic stilbenoids and TRPA1 should be thoroughly investigated in oncological settings to develop novel adjuvant therapeutics targeting TRPA1-expressing cancers.

## Figures and Tables

**Table 1:** Clinical trials registered with results for stilbene compounds.

Compound	Code	Title	Details
Resveratrol	NCT00920556	A Clinical Study to Assess the Safety and Activity of SRT501 Alone or in Combination With Bortezomib in Patients With Multiple Myeloma	5 g of SRT501 were administered for 20 consecutive days in a 21-day cycle
Resveratrol	NCT01354977	Effect of Resveratrol on Age-related Insulin Resistance and Inflammation in Humans	Two 500 mg capsules administered twice a day for 28 days
Resveratrol	NCT04400890	Randomized Proof-of-Concept Trial to Evaluate the Safety and Explore the Effectiveness of Resveratrol, a Plant Polyphenol, for COVID-19	1 g administered 4 times a day for a minimum of 7 days
Resveratrol	NCT03866200	Resveratrol Trial for Relief of Pain in Pseudoachondroplasia	125 mg/day for 90 days
Resveratrol	NCT03253913	Resveratrol and Sirolimus in Lymphangioleiomyomatosis Trial	250 mg/day for the first 8 weeks, followed by 250 mg twice daily for the next 8 weeks, and then 500 mg twice daily for the last 8 weeks
Resveratrol	NCT01375959	Pilot Study of Resveratrol in Older Adults With Impaired Glucose Tolerance (RSV)	1.5 g twice a day for 6 weeks
Resveratrol	NCT02523274	Resveratrol and Exercise to Treat Functional Limitations in Late Life	Exercise and 500 mg/day or 1000 mg/day resveratrol
Pterostilbene	NCT01267227	Effect of Pterostilbene on Cholesterol, Blood Pressure and Oxidative Stress	50 mg or 125 mg twice daily

## Data Availability

Not applicable.

## Supplementary Materials

The following supporting information can be downloaded at: https://www.mdpi.com/article/10.3390/plants12162935/s1, Table S1: Representative families and genera of plants containing oligomeric stilbenoid compounds. Refs. [112,113,114,115,116,117,118] are cited in Supplementary Materials.
