# Supplementary material for: Resveratrol and Other Natural Oligomeric Stilbenoid Compounds and Their Therapeutic Applications
Source: Plants (Basel). 2023 Aug 14;12(16):2935. doi: 10.3390/plants12162935 (PMC10459741; doi:10.3390/plants12162935)
Supplement: Supplementary file 1 [file plants-12-02935-s001.zip › plants-2525278-SI.pdf]

plants MDPI

Review

Resveratrol and Other Natural Oligomeric Stilbenoid Compounds and Their Therapeutic Applications

Cosmina-Gabriela Duta-Bratu, George Mihai Nitulescu *, Dragos Paul Mihai * and Octavian Tudorel Olaru

Faculty of Pharmacy, "Carol Davila" University of Medicine and Pharmacy, Traian Vuia 6, 020956 Bucharest, Romania

Supplementary Material

Table S1. Representative families and genera of plants containing oligomeric stilbenoid compounds.

Plant Family	Representative Genus
Gnetaceae [1,2]	*Gnetum*
Pinaceae [3,4]	*Pinus*
	Picea
Moraceae [5,6]	*Morus*
	Artocarpus
Polygonaceae [7][8]	*Rheum*
	Polygonum
Euphorbiaceae [9–11]	*Macaranga*
Vitaceae [1,8]	*Vitis*
	Ampelopsis
	Cayratia
	Cissus
	Cyphostemma
	Rhoicissus
	Muscadinia
	Parthenocissus
Fabaceae [12]	*Lonchocarpus*
	Pterocarpus
Ericaceae [13]	*Vaccinium*
	Gaylussacia
Cyperaceae [14]	*Carex*
	Cyperus
	Carpha
	Kobresia
	Scirpus

References

1. Rivière, C.; Pawlus, A. D.; Mérillon, J. M. Natural stilbenoids: Distribution in the plant kingdom and chemotaxonomic interest in Vitaceae. *Nat. Prod. Rep.* **2012**, *29*, 1317–1333.
2. Teka, T.; Zhang, L.; Ge, X.; Li, Y.; Han, L.; Yan, X. Stilbenes: Source plants, chemistry, biosynthesis, pharmacology, application and problems related to their clinical Application-A comprehensive review. *Phytochemistry* **2022**, *197*, 113128.
3. Valletta, A.; Iozia, L. M.; Leonelli, F. Impact of Environmental Factors on Stilbene Biosynthesis. *Plants* **2021**, *10*.
4. Jyske, T.; Brännström, H.; Halmemies, E.; Laakso, T.; Kilpeläinen, P.; Hyvönen, J.; Kärkkäinen, K.; Saranpää, P. Stilbenoids of Norway spruce bark: does the variability caused by raw-material processing offset the biological variability? *Biomass Convers. Biorefinery* **2022**.

5. Arslan, G.; Yilmaz, N. Determination of Trans-resveratrol levels in different fruits, vegetables and their skin by HPLC. *Asian J. Chem.* **2013**, *25*, 1225–1228.
6. Boonlaksiri, C.; Oonanant, W.; Kongsaeree, P.; Kittakoop, P.; Tanticharoen, M.; Thebtaranonth, Y. An antimalarial stilbene from Artocarpus integer. *Phytochemistry* **2000**, *54*, 415–417.
7. Zheng, Q.; Wu, H.; Guo, J.; Nan, H.; Chen, S.; Yang, J.; Xu, X. Review of Rhubarbs: Chemistry and Pharmacology. *Chinese Herb. Med.* **2013**, *5*, 9–32.
8. Su, X.; Zhou, D.; Li, N. Chapter 8 - Bioactive stilbenes from plants. In; Atta-ur-Rahman, B. T.-S. in N. P. C., Ed.; Elsevier, 2022; Vol. 73, pp. 265–403.
9. van der Kaaden, J. E.; Hemscheidt, T. K.; Mooberry, S. L. Mappain, a New Cytotoxic Prenylated Stilbene from Macaranga mappa. *J. Nat. Prod.* **2001**, *64*, 103–105.
10. Jang, D. S.; Cuendet, M.; Hawthorne, M. E.; Kardono, L. B. S.; Kawanishi, K.; Fonga, H. H. S.; Mehta, R. G.; Pezzuto, J. M.; Kinghorn, A. D. Prenylated flavonoids of the leaves of Macaranga conifera with inhibitory activity against cyclooxygenase-2. *Phytochemistry* **2002**, *61*, 867–872.
11. Beutler, J. A.; Jato, J.; Cragg, G. M.; Boyd, M. R. Schweinfurthin D, a cytotoxic stilbene from Macaranga schweinfurthii. *Nat. Prod. Lett.* **2000**, *14*, 399–404.
12. Ioset, J.-R.; Marston, A.; Gupta, M. P.; Hostettmann, K. Five New Prenylated Stilbenes from the Root Bark of Lonchocarpus chiricanus. *J. Nat. Prod.* **2001**, *64*, 710–715.
13. Liu, Y.; You, Y.; Lu, J.; Chen, X.; Yang, Z. Recent Advances in Synthesis, Bioactivity, and Pharmacokinetics of Pterostilbene, an Important Analog of Resveratrol. *Molecules* **2020**, *25*.
14. Dávid, C. Z.; Hohmann, J.; Vasas, A. Chemistry and Pharmacology of Cyperaceae Stilbenoids: A Review. *Molecules* **2021**, *26*.

Disclaimer/Publisher's Note: The statements, opinions and data contained in all publications are solely those of the individual author(s) and contributor(s) and not of MDPI and/or the editor(s). MDPI and/or the editor(s) disclaim responsibility for any injury to people or property resulting from any ideas, methods, instructions or products referred to in the content.
